# Redetermination of AgNb_2_PS_10_ revealing a silver deficiency

**DOI:** 10.1107/S1600536809025100

**Published:** 2009-07-04

**Authors:** Junghwan Do, Hoseop Yun

**Affiliations:** a Department of Chemistry, Konkuk University, Seoul 143-701, Republic of Korea; b Division of Energy Systems Research and Department of Chemistry, Ajou University, Suwon 443-749, Republic of Korea

## Abstract

In comparison with a previous crystallographic study [Goh *et al.* (2002 ▶). *J. Solid State Chem.* **168**, 119–125] of the title compound, silver diniobium tris­(disulfide) tetra­thio­phosphate(V), that reports a full occupation of the silver position and isotropic displacement parameters for the atoms, the current redetermination reveals a silver deficiency with a site-occupation factor of 0.88 (1) and reports all atoms with anisotropic displacement parameters. The structure of Ag_0.88_Nb_2_PS_10_ is composed of _∞_^1^[Nb_2_PS_10_] chains, which are built up from pairs of distorted bicapped trigonal-prismatic [NbS_8_] polyhedra forming [Nb_2_S_12_] dimers and of tetra­hedral [PS_4_] groups. These chains are connected *via* the statistically disordered Ag^+^ ions, forming double layers. Adjacent layers are stacked solely through van der Waals forces into a three-dimensional structure. Short and long Nb—Nb distances [2.880 (1) and 3.770 (2) Å, respectively] alternate along the chain and S_2_^2−^ and S^2−^ anionic species are observed.

## Related literature

The synthesis and structural characterization of stoichiometric AgNb_2_PS_10_ and NaNb_2_PS_10_ have been published (Goh *et al., *2002). For Nb_2_PS_10_-related quaternary thio­phosphates with general formula *M*Nb_2_PS_10_, see: Do & Yun (1996 ▶) for KNb_2_PS_10_, Kim & Yun (2002 ▶) for RbNb_2_PS_10_, Kwak *et al.* (2007 ▶) for CsNb_2_PS_10_, and Bang *et al.* (2008 ▶) for TlNb_2_PS_10_; for related penta­nary thio­phosphates *M,M′*Nb_2_PS_10_, see: Kwak & Yun (2008 ▶) for K_0.34_Cu_0.5_Nb_2_PS_10_, Dong *et al.* (2005*a* ▶) for K_0.5_Ag_0.5_Nb_2_PS_10_, and Dong *et al.* (2005*b* ▶) for Rb_0.38_Ag_0.5_Nb_2_PS_10_. For data standardization, see: Gelato & Parthé (1987 ▶). For ionic radii, see: Shannon (1976 ▶). For structure validation, see: Spek (2009 ▶). For typical P—S bond distances, see: Brec *et al.* (1983 ▶). For typical Nb^4+^—Nb^4+^ bond distances, see: Angenault *et al.* (2000 ▶).

## Experimental

### 

#### Crystal data

Ag_0.88_Nb_2_PS_10_
*M* *_r_* = 631.78
Monoclinic, 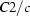

*a* = 24.001 (5) Å
*b* = 7.7711 (17) Å
*c* = 12.960 (3) Å
β = 94.833 (19)°
*V* = 2408.6 (9) Å^3^
*Z* = 8
Mo *K*α radiation
μ = 5.1 mm^−1^
*T* = 290 K
0.60 × 0.06 × 0.04 mm

#### Data collection

MAC Science MXC3 diffractometer
Absorption correction: analytical (de Meulenaer & Tompa, 1965 ▶) *T*_min_ = 0.727, *T*_max_ = 0.821
2221 measured reflections
2114 independent reflections
1835 reflections with *I* > 2σ(*I*)
*R*_int_ = 0.017
2 standard reflections every 100 reflections intensity decay: none

#### Refinement

*R*[*F* ^2^ > 2σ(*F* ^2^)] = 0.045
*wR*(*F* ^2^) = 0.109
*S* = 1.16
2114 reflections
128 parameters
Δρ_max_ = 1.82 e Å^−3^
Δρ_min_ = −1.20 e Å^−3^

### 

Data collection: *MAC Science MXC3* (MAC Science, 1994 ▶); cell refinement: *MAC Science MXC3*; data reduction: *MAC Science MXC3*; program(s) used to solve structure: *SHELXS97* (Sheldrick, 2008 ▶); program(s) used to refine structure: *SHELXL97* (Sheldrick, 2008 ▶); molecular graphics: locally modified version of *ORTEP* (Johnson, 1965 ▶); software used to prepare material for publication: *WinGX* (Farrugia, 1999 ▶).

## Supplementary Material

Crystal structure: contains datablocks global, I. DOI: 10.1107/S1600536809025100/wm2240sup1.cif

Structure factors: contains datablocks I. DOI: 10.1107/S1600536809025100/wm2240Isup2.hkl

Additional supplementary materials:  crystallographic information; 3D view; checkCIF report

## Figures and Tables

**Table:** 

Ag—S1^i^	2.536 (3)
Ag—S9^ii^	2.620 (3)
Ag—S2^iii^	2.875 (3)
Ag—S8^iv^	2.916 (3)
Ag—S1^iii^	2.965 (4)
Ag—S3	3.091 (3)
Nb1—S5	2.462 (2)
Nb1—S2^v^	2.466 (2)
Nb1—S7^vi^	2.518 (3)
Nb1—S6^iv^	2.551 (2)
Nb1—S3	2.554 (3)
Nb1—S4^v^	2.562 (2)
Nb1—S8^iv^	2.573 (3)
Nb1—S10^iv^	2.659 (2)
Nb2—S3^ii^	2.476 (3)
Nb2—S7	2.479 (3)
Nb2—S5^ii^	2.508 (2)
Nb2—S2^vii^	2.551 (3)
Nb2—S4^ii^	2.558 (2)
Nb2—S6	2.569 (3)
Nb2—S9	2.630 (3)
Nb2—S10	2.656 (2)
P—S1^vi^	2.009 (4)
P—S8	2.048 (4)
P—S9	2.059 (4)
P—S10	2.065 (3)

**Table:** 

S1^vi^—P—S8	108.46 (17)
S1^vi^—P—S9	112.81 (17)
S8—P—S9	111.87 (16)
S1^vi^—P—S10	117.65 (16)
S8—P—S10	104.24 (14)
S9—P—S10	101.46 (14)

Symmetry codes: (i) 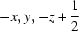; (ii) 
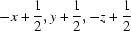
; (iii) 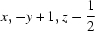; (iv) 
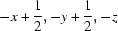
; (v) 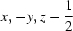; (vi) 
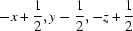
; (vii) 
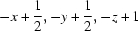
.

**Fig. 1.**
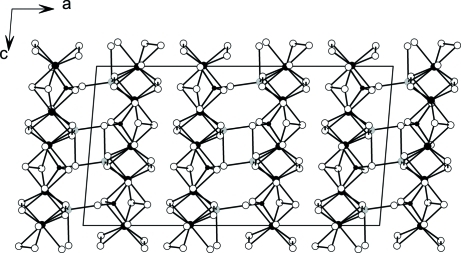
A view of the structure of Ag0.88Nb2PS10 down the b axis showing the double layers and the two-dimensional nature of the compound. Large and small filled circles are Nb and P atoms respectively; large open circles are S atoms; grey circles represent Ag atoms.

**Fig. 2.**
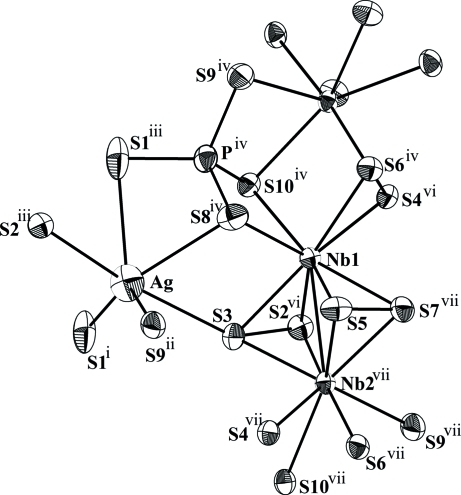
A view of the structure of Ag0.88Nb2PS10 showing the coordination around Nb, Ag, and P atoms. Anisotropic displacement ellipsoids are drawn at the 70% probability level. Symmetry codes are as given in Table 1.

## supplementary crystallographic information

### Comment

In comparison with the previous study of AgNb_2_PS_10_ (Goh *et al.*, 2002), both lattice parameters and atomic coordinates of the current redetermination are the same within their standard deviations. However, our investigation indicated that there is a deficiency of Ag atoms in the title compound with a site occupation factor (s.o.f.) of 0.88 (1). This observation is consistent with crystal structure refinements from crystals obtained from other reaction batches. Therefore we assume that the crystal originally investigated by Goh *et al.* (2002) shows the same behaviour. In general, non-stoichiometry in multinary niobium  thiophosphates is not uncommon and has been observed in one of our previous studies (Kwak & Yun, 2008).

The structure of Ag_0.88_Nb_2_PS_10_ consists of one-dimensional _∞_^1^[Nb_2_PS_10_] chains along the [001] direction that are connected *via* the statistically disordered Ag^+^ ions to form a double layer parallel to the *bc* plane. These layers then stack on top of each other to form the three-dimensional structure with a van der Waals gap as shown in Fig. 1. There is no bonding interaction, only van der Waals forces, between the double layers.

As shown in other phases in the *M,M'*Nb_2_PS_10_ family, *viz* KNb_2_PS_10_ (Do & Yun, 1996), RbNb_2_PS_10 _(Kim & Yun, 2002), CsNb_2_PS_10_ (Kwak *et al.,* 2007), TlNb_2_PS_10_ (Bang *et al.,* 2008), K_0.34_Cu_0.5_Nb_2_PS_10_ (Kwak & Yun, 2008), K_0.5_Ag_0.5_Nb_2_PS_10_ (Dong *et al.,* 2005*a*), and Rb_0.38_Ag_0.5_Nb_2_PS_10_ (Dong *et al.*, 2005*b*), each of the chains is made up of pairs of [NbS_8_] polyhedra forming characteristic [Nb_2_S_12_] units and tetrahedral [PS_4_] groups. In the title compound, the Nb1 and Nb2 atoms are surrounded by 8 S atoms in a bicapped trigonal-prismatic fashion. Two prisms are sharing a rectangular face to form the [Nb_2_S_12_] unit. This unit shows an approximate 2-fold rotational symmetry and the rotation axis bisects the short Nb1—Nb2 distance and the (S—S)^2-^ sides of the rectangular face shared by each trigonal prism. The [Nb_2_S_12_] unit is bound to each other to form the infinite [Nb_2_S_9_] chains by sharing the S—S prism edge. One of the S atoms at the prism edge and two other capping S atoms are bound to the P atom and an additional S atom (S1) is attached to the P atom to complete the [PS_4_] tetrahedral coordination. The P—S distances (2.048 (4)–2.065 (3) Å) are in good agreement with P—S distances found in related phases (Brec *et al.*, 1983). The S1 atom is the only sulfur atom that is not coordinated to any of the Nb atoms causing the short P—S1 distance (2.009 (4) Å) as well as the large ADP of the S1 atom (Do & Yun, 1996). Along the chains, the Nb atoms associate in pairs with Nb—Nb interactions alternating in the sequence of one short and one long distances. Although the short distance (2.880 (1) Å) is typical of Nb^4+^—Nb^4+^ bonding interactions (Angenault *et al.*, 2000), the long distance (3.770 (2) Å) implies that there is no significant Nb—Nb interaction and such an arrangement is consistent with the highly resistive and diamagnetic nature of the compound.

The silver atom is surrounded by six S atoms. The coordination around the Ag atom can be described as [2 + 4]  (Fig. 2). Two S atoms are coordinated to the Ag atom (Ag—S1, 2.536 (3) Å; Ag—S9, 2.620 (3) Å), whereas four S atoms are weakly bound to the Ag atoms (Ag—S, 2.875 (3)–3.091 (3) Å). These distances are comparable to the sum of the ionic radii of each element, 2.51 Å for CN=2 and 2.99 Å for CN=6 (Shannon, 1976).

### Experimental

Ag_0.88_Nb_2_PS_10_ was prepared by the reaction of the elements Nb, P, and S with an elemental ratio of 2:1:10 in the eutectic mixture of AgCl/LiCl (Kojima, 99.5%). The starting materials, Nb powder (CERAC 99.8%), P powder (CERAC 99.5%), and S powder (Aldrich 99.999%) were placed in a silica glass tube. The mass ratio of reactants and halide flux was 1:2. The tube was evacuated to 0.133 Pa, sealed, and heated to 973 K where it was kept for 7d. Afterwards, the tube was cooled at a rate of 4 K/h to room temperature. Black needle-shaped crystals were isolated from the flux by leaching out with water. The crystals are stable in water and air. Electron microprobe analysis of the crystals established their homogeneity and the presence of Ag, Nb, P and S. No other element was detected.

### Refinement

Large anisotropic displacement parameters (ADPs) of the silver atom were found when the structure was refined with the stoichiometric model AgNb_2_PS_10_, (Goh *et al., *2002). The deficient nature of the Ag site was checked by refining the occupancy of Ag while that of the other atoms were fixed. With the non-stoichiometric model (Ag*_x_*Nb_2_PS_10_), the occupation factor of the Ag site was reduced significantly from 1 to 0.88 (1) and the reliability factor (wR2 = 0.1089) was improved in comparison with full occupation of the silver position (wR2 = 0.1341). In addition, the anisotropic displacement parameters in the disordered model became plausible. As no evidence was found for ordering of the Ag site, a statistically disordered structure was assumed. With the composition established, the data for the compound were corrected for absorption with the use of the analytical method (de Meulenaer & Tompa, 1965). The highest residual electron density is 0.96 Å from the S1 site and the deepest hole is 0.73 Å from the Ag site. No additional symmetry, as tested by *PLATON* (Spek, 2009), was detected in this structure. Structure data were finally standardized by means of the program *STRUCTURE TIDY* (Gelato & Parthé, 1987).

### Crystal data

**Table:**

Ag_0.88_Nb_2_PS_10_	*F*(000) = 2385
*M**_r_* = 631.78	*D*_x_ = 3.485 Mg m^−^^3^
Monoclinic, *C*2/*c*	Mo *K*α radiation, λ = 0.71073 Å
Hall symbol: -C 2yc	Cell parameters from 24 reflections
*a* = 24.001 (5) Å	θ = 10.0–15.0°
*b* = 7.7711 (17) Å	µ = 5.1 mm^−^^1^
*c* = 12.960 (3) Å	*T* = 290 K
β = 94.833 (19)°	Needle, black
*V* = 2408.6 (9) Å^3^	0.60 × 0.06 × 0.04 mm
*Z* = 8	

### Data collection

**Table:**

MAC Science MXC3 diffractometer	1835 reflections with *I* > 2σ(*I*)
Radiation source: normal-focus sealed tube	*R*_int_ = 0.017
graphite	θ_max_ = 25.0°, θ_min_ = 1.7°
ω–2θ scans	*h* = −28→28
Absorption correction: analytical (de Meulenaer & Tompa, 1965)	*k* = 0→9
*T*_min_ = 0.727, *T*_max_ = 0.821	*l* = 0→15
2221 measured reflections	2 standard reflections every 100 reflections
2114 independent reflections	intensity decay: none

### Refinement

**Table:**

Refinement on *F*^2^	0 restraints
Least-squares matrix: full	Primary atom site location: structure-invariant direct methods
*R*[*F*^2^ > 2σ(*F*^2^)] = 0.045	Secondary atom site location: difference Fourier map
*wR*(*F*^2^) = 0.109	*w* = 1/[σ^2^(*F*_o_^2^) + (0.029*P*)^2^ + 98.0899*P*] where *P* = (*F*_o_^2^ + 2*F*_c_^2^)/3
*S* = 1.16	(Δ/σ)_max_ < 0.001
2114 reflections	Δρ_max_ = 1.82 e Å^−^^3^
128 parameters	Δρ_min_ = −1.19 e Å^−^^3^

### Special details

**Table:**

Geometry. All s.u.'s (except the s.u. in the dihedral angle between two l.s. planes) are estimated using the full covariance matrix. The cell s.u.'s are taken into account individually in the estimation of s.u.'s in distances, angles and torsion angles; correlations between s.u.'s in cell parameters are only used when they are defined by crystal symmetry. An approximate (isotropic) treatment of cell s.u.'s is used for estimating s.u.'s involving l.s. planes.
Refinement. Refinement of *F*^2^ against ALL reflections. The weighted *R*-factor *wR* and goodness of fit *S* are based on *F*^2^, conventional *R*-factors *R* are based on *F*, with *F* set to zero for negative *F*^2^. The threshold expression of *F*^2^ > 2σ(*F*^2^) is used only for calculating *R*-factors(gt) *etc*. and is not relevant to the choice of reflections for refinement. *R*-factors based on *F*^2^ are statistically about twice as large as those based on *F*, and *R*- factors based on ALL data will be even larger.

### Fractional atomic coordinates and isotropic or equivalent isotropic displacement parameters (Å^2^)

**Table:**

	*x*	*y*	*z*	*U*_iso_*/*U*_eq_	Occ. (<1)
Ag	0.05548 (4)	0.51279 (13)	0.09923 (9)	0.0397 (4)	0.878 (4)
Nb1	0.13913 (3)	0.05530 (10)	0.00408 (6)	0.0156 (2)	
Nb2	0.36226 (3)	0.43048 (10)	0.28691 (6)	0.0155 (2)	
P	0.40291 (10)	0.1092 (3)	0.1376 (2)	0.0223 (5)	
S1	0.04299 (12)	0.4143 (4)	0.3739 (3)	0.0399 (7)	
S2	0.06326 (9)	0.1230 (3)	0.56424 (18)	0.0208 (5)	
S3	0.06437 (10)	0.1186 (3)	0.12793 (19)	0.0240 (5)	
S4	0.15627 (9)	0.1535 (3)	0.35711 (17)	0.0186 (5)	
S5	0.21371 (10)	0.1063 (3)	0.14306 (18)	0.0243 (5)	
S6	0.28921 (9)	0.4481 (3)	0.13057 (18)	0.0204 (5)	
S7	0.28941 (10)	0.3584 (3)	0.40463 (19)	0.0245 (5)	
S8	0.34993 (11)	0.1158 (3)	0.00528 (19)	0.0276 (6)	
S9	0.36001 (11)	0.0935 (3)	0.2684 (2)	0.0257 (5)	
S10	0.43518 (9)	0.3553 (3)	0.15040 (17)	0.0185 (5)	

### Atomic displacement parameters (Å^2^)

**Table:**

	*U*^11^	*U*^22^	*U*^33^	*U*^12^	*U*^13^	*U*^23^
Ag	0.0377 (6)	0.0321 (6)	0.0492 (7)	−0.0076 (4)	0.0037 (5)	−0.0051 (5)
Nb1	0.0138 (4)	0.0169 (4)	0.0160 (4)	−0.0008 (3)	0.0009 (3)	0.0006 (3)
Nb2	0.0131 (4)	0.0155 (4)	0.0178 (4)	0.0003 (3)	0.0014 (3)	0.0006 (3)
P	0.0228 (13)	0.0150 (12)	0.0295 (13)	−0.0008 (10)	0.0049 (10)	0.0013 (10)
S1	0.0326 (15)	0.0216 (13)	0.066 (2)	−0.0078 (12)	0.0070 (14)	0.0056 (13)
S2	0.0156 (11)	0.0233 (12)	0.0234 (12)	0.0028 (9)	0.0012 (9)	−0.0009 (10)
S3	0.0205 (12)	0.0256 (13)	0.0260 (12)	0.0066 (10)	0.0025 (9)	0.0025 (10)
S4	0.0193 (11)	0.0152 (11)	0.0212 (11)	−0.0010 (9)	0.0010 (9)	−0.0012 (9)
S5	0.0189 (11)	0.0324 (14)	0.0211 (12)	−0.0080 (10)	−0.0006 (9)	0.0029 (10)
S6	0.0148 (11)	0.0253 (12)	0.0209 (11)	−0.0024 (9)	0.0011 (9)	0.0001 (9)
S7	0.0200 (12)	0.0309 (13)	0.0230 (12)	−0.0063 (10)	0.0034 (9)	−0.0016 (10)
S8	0.0395 (15)	0.0215 (13)	0.0210 (12)	−0.0060 (11)	−0.0016 (11)	−0.0024 (10)
S9	0.0313 (13)	0.0191 (12)	0.0274 (13)	−0.0017 (10)	0.0068 (10)	0.0018 (10)
S10	0.0156 (10)	0.0182 (11)	0.0214 (12)	−0.0005 (9)	0.0004 (9)	0.0022 (9)

### Geometric parameters (Å, °)

**Table:**

Ag—S1^i^	2.536 (3)	Nb2—S3^ii^	2.476 (3)
Ag—S9^ii^	2.620 (3)	Nb2—S7	2.479 (3)
Ag—S2^iii^	2.875 (3)	Nb2—S5^ii^	2.508 (2)
Ag—S8^iv^	2.916 (3)	Nb2—S2^viii^	2.551 (3)
Ag—S1^iii^	2.965 (4)	Nb2—S4^ii^	2.558 (2)
Ag—S3	3.091 (3)	Nb2—S6	2.569 (3)
Ag—P^iv^	3.440 (3)	Nb2—S9	2.630 (3)
Ag—Ag^v^	3.549 (2)	Nb2—S10	2.656 (2)
Ag—P^ii^	3.552 (3)	Nb1—Nb2^vii^	2.8800 (13)
Ag—Nb1	4.3137 (15)	Nb1—P^iv^	3.298 (3)
Nb1—S5	2.462 (2)	Nb1—Nb2^iv^	3.7702 (15)
Nb1—S2^vi^	2.466 (2)	Nb2—Nb1^ii^	2.8800 (13)
Nb1—S7^vii^	2.518 (3)	Nb2—Nb1^iv^	3.7702 (15)
Nb1—S6^iv^	2.551 (2)	P—S1^vii^	2.009 (4)
Nb1—S3	2.554 (3)	P—S8	2.048 (4)
Nb1—S4^vi^	2.562 (2)	P—S9	2.059 (4)
Nb1—S8^iv^	2.573 (3)	P—S10	2.065 (3)
Nb1—S10^iv^	2.659 (2)		
			
S1^i^—Ag—S9^ii^	131.39 (11)	S7—Nb2—S5^ii^	47.83 (9)
S1^i^—Ag—S2^iii^	113.11 (9)	S3^ii^—Nb2—S2^viii^	48.11 (8)
S9^ii^—Ag—S2^iii^	79.08 (8)	S7—Nb2—S2^viii^	89.05 (8)
S1^i^—Ag—S8^iv^	136.99 (9)	S5^ii^—Nb2—S2^viii^	107.42 (8)
S9^ii^—Ag—S8^iv^	78.25 (8)	S3^ii^—Nb2—S4^ii^	89.92 (8)
S2^iii^—Ag—S8^iv^	101.58 (7)	S7—Nb2—S4^ii^	120.91 (8)
S1^i^—Ag—S1^iii^	100.08 (10)	S5^ii^—Nb2—S4^ii^	78.94 (8)
S9^ii^—Ag—S1^iii^	127.42 (9)	S2^viii^—Nb2—S4^ii^	136.85 (8)
S2^iii^—Ag—S1^iii^	70.06 (8)	S3^ii^—Nb2—S6	137.11 (9)
S8^iv^—Ag—S1^iii^	68.05 (8)	S7—Nb2—S6	91.58 (8)
S1^i^—Ag—S3	74.93 (8)	S5^ii^—Nb2—S6	77.74 (8)
S9^ii^—Ag—S3	96.70 (8)	S2^viii^—Nb2—S6	173.34 (8)
S2^iii^—Ag—S3	171.84 (8)	S4^ii^—Nb2—S6	47.42 (8)
S8^iv^—Ag—S3	70.59 (7)	S3^ii^—Nb2—S9	129.58 (9)
S1^iii^—Ag—S3	107.95 (8)	S7—Nb2—S9	79.70 (8)
S1^i^—Ag—P^iv^	112.54 (9)	S5^ii^—Nb2—S9	124.43 (9)
S9^ii^—Ag—P^iv^	112.55 (8)	S2^viii^—Nb2—S9	85.10 (8)
S2^iii^—Ag—P^iv^	95.91 (7)	S4^ii^—Nb2—S9	127.37 (8)
S8^iv^—Ag—P^iv^	36.42 (7)	S6—Nb2—S9	88.49 (8)
S1^iii^—Ag—P^iv^	35.58 (7)	S3^ii^—Nb2—S10	86.73 (8)
S3—Ag—P^iv^	79.17 (7)	S7—Nb2—S10	153.96 (9)
S1^i^—Ag—Ag^v^	55.36 (8)	S5^ii^—Nb2—S10	154.21 (8)
S9^ii^—Ag—Ag^v^	168.61 (8)	S2^viii^—Nb2—S10	90.51 (8)
S2^iii^—Ag—Ag^v^	89.75 (6)	S4^ii^—Nb2—S10	75.33 (7)
S8^iv^—Ag—Ag^v^	102.14 (7)	S6—Nb2—S10	86.02 (8)
S1^iii^—Ag—Ag^v^	44.72 (6)	S9—Nb2—S10	74.33 (8)
S3—Ag—Ag^v^	94.11 (6)	S3^ii^—Nb2—Nb1^ii^	56.36 (6)
S5—Nb1—S2^vi^	111.70 (8)	S7—Nb2—Nb1^ii^	55.44 (6)
S5—Nb1—S7^vii^	47.90 (9)	S5^ii^—Nb2—Nb1^ii^	53.84 (6)
S2^vi^—Nb1—S7^vii^	90.11 (8)	S2^viii^—Nb2—Nb1^ii^	53.60 (6)
S5—Nb1—S6^iv^	90.67 (8)	S4^ii^—Nb2—Nb1^ii^	116.29 (6)
S2^vi^—Nb1—S6^iv^	140.23 (9)	S6—Nb2—Nb1^ii^	131.50 (6)
S7^vii^—Nb1—S6^iv^	80.99 (8)	S9—Nb2—Nb1^ii^	114.81 (6)
S5—Nb1—S3	90.87 (8)	S10—Nb2—Nb1^ii^	139.55 (6)
S2^vi^—Nb1—S3	48.16 (8)	S3^ii^—Nb2—Nb1^iv^	112.00 (6)
S7^vii^—Nb1—S3	107.97 (8)	S7—Nb2—Nb1^iv^	132.44 (6)
S6^iv^—Nb1—S3	168.99 (8)	S5^ii^—Nb2—Nb1^iv^	113.61 (6)
S5—Nb1—S4^vi^	119.57 (9)	S2^viii^—Nb2—Nb1^iv^	135.05 (6)
S2^vi^—Nb1—S4^vi^	92.77 (8)	S4^ii^—Nb2—Nb1^iv^	42.61 (5)
S7^vii^—Nb1—S4^vi^	79.56 (8)	S6—Nb2—Nb1^iv^	42.40 (5)
S6^iv^—Nb1—S4^vi^	47.56 (8)	S9—Nb2—Nb1^iv^	86.53 (6)
S3—Nb1—S4^vi^	138.99 (8)	S10—Nb2—Nb1^iv^	44.85 (5)
S5—Nb1—S8^iv^	78.70 (9)	Nb1^ii^—Nb2—Nb1^iv^	158.61 (3)
S2^vi^—Nb1—S8^iv^	130.83 (9)	S1^vii^—P—S8	108.46 (17)
S7^vii^—Nb1—S8^iv^	123.89 (9)	S1^vii^—P—S9	112.81 (17)
S6^iv^—Nb1—S8^iv^	84.30 (8)	S8—P—S9	111.87 (16)
S3—Nb1—S8^iv^	85.31 (9)	S1^vii^—P—S10	117.65 (16)
S4^vi^—Nb1—S8^iv^	124.80 (8)	S8—P—S10	104.24 (14)
S5—Nb1—S10^iv^	155.38 (9)	S9—P—S10	101.46 (14)
S2^vi^—Nb1—S10^iv^	85.32 (8)	Nb1^ix^—S2—Nb2^viii^	70.04 (7)
S7^vii^—Nb1—S10^iv^	154.11 (8)	S3^ix^—S2—Ag^x^	146.81 (13)
S6^iv^—Nb1—S10^iv^	86.32 (8)	S2^vi^—S3—Nb2^vii^	67.87 (10)
S3—Nb1—S10^iv^	87.75 (8)	S2^vi^—S3—Nb1	63.68 (9)
S4^vi^—Nb1—S10^iv^	75.23 (7)	Nb2^vii^—S3—Nb1	69.84 (7)
S8^iv^—Nb1—S10^iv^	76.69 (8)	S2^vi^—S3—Ag	149.39 (13)
S5—Nb1—Nb2^vii^	55.35 (6)	Nb2^vii^—S3—Ag	132.70 (10)
S2^vi^—Nb1—Nb2^vii^	56.36 (6)	Nb1—S3—Ag	99.22 (8)
S7^vii^—Nb1—Nb2^vii^	54.18 (6)	S6^vii^—S4—Nb2^vii^	66.56 (9)
S6^iv^—Nb1—Nb2^vii^	134.56 (6)	S6^vii^—S4—Nb1^ix^	65.96 (9)
S3—Nb1—Nb2^vii^	53.80 (6)	Nb2^vii^—S4—Nb1^ix^	94.84 (8)
S4^vi^—Nb1—Nb2^vii^	120.09 (6)	S7^vii^—S5—Nb1	67.50 (10)
S8^iv^—Nb1—Nb2^vii^	112.84 (6)	S7^vii^—S5—Nb2^vii^	65.33 (10)
S10^iv^—Nb1—Nb2^vii^	137.39 (6)	Nb1—S5—Nb2^vii^	70.81 (7)
S5—Nb1—Nb2^iv^	132.20 (6)	S4^ii^—S6—Nb1^iv^	66.48 (9)
S2^vi^—Nb1—Nb2^iv^	112.84 (6)	S4^ii^—S6—Nb2	66.02 (9)
S7^vii^—Nb1—Nb2^iv^	115.85 (6)	Nb1^iv^—S6—Nb2	94.83 (8)
S6^iv^—Nb1—Nb2^iv^	42.76 (6)	S5^ii^—S7—Nb2	66.84 (10)
S3—Nb1—Nb2^iv^	132.49 (6)	S5^ii^—S7—Nb1^ii^	64.60 (10)
S4^vi^—Nb1—Nb2^iv^	42.55 (5)	Nb2—S7—Nb1^ii^	70.38 (7)
S8^iv^—Nb1—Nb2^iv^	85.19 (6)	P—S8—Nb1^iv^	90.34 (11)
S10^iv^—Nb1—Nb2^iv^	44.79 (5)	P—S9—Nb2	90.49 (11)
Nb2^vii^—Nb1—Nb2^iv^	161.96 (3)	P—S10—Nb2	89.62 (11)
P^iv^—Nb1—Nb2^iv^	56.18 (5)	P—S10—Nb1^iv^	87.61 (11)
S3^ii^—Nb2—S7	111.78 (8)	Nb2—S10—Nb1^iv^	90.36 (7)
S3^ii^—Nb2—S5^ii^	91.64 (9)		

Symmetry codes: (i) −*x*, *y*, −*z*+1/2; (ii) −*x*+1/2, *y*+1/2, −*z*+1/2; (iii) *x*, −*y*+1, *z*−1/2; (iv) −*x*+1/2, −*y*+1/2, −*z*; (v) −*x*, −*y*+1, −*z*; (vi) *x*, −*y*, *z*−1/2; (vii) −*x*+1/2, *y*−1/2, −*z*+1/2; (viii) −*x*+1/2, −*y*+1/2, −*z*+1; (ix) *x*, −*y*, *z*+1/2; (x) *x*, −*y*+1, *z*+1/2.
